# *oip*A “on” status of *Helicobacter pylori* is associated with gastric cancer in North-Eastern Brazil

**DOI:** 10.1186/s12885-018-5249-x

**Published:** 2019-01-10

**Authors:** Lúcia Libanez Bessa Campelo Braga, Maria Helane Rocha Batista, Orleâncio Gomes Ripardo de Azevedo, Kassiane Cristine da Silva Costa, Adriana Dias Gomes, Gifone Aguiar Rocha, Dulciene Maria Magalhães Queiroz

**Affiliations:** 1 Clinical Research Unit, University Hospital Walter Cantídio/Department of Internal Medicine, Universidade Federal do Ceará, Fortaleza, Brazil; 2 Institute of Biomedicine, Universidade Federal do Ceará, Fortaleza, Brazil; 3 Laboratory of Research in Bacteriology, Faculdade de Medicina, Universidade Federal de Minas Gerais, Av. Alfredo Balena, 190 – Sala 216, Belo Horizonte, CEP: 30130-100 Brazil

**Keywords:** *Helicobacter pylori*, *oip*A, *cag*A, *vac*A, Gastric cancer

## Abstract

**Background:**

Although, outer membrane protein OipA of *Helicobacter pylori* has been associated with gastric mucosal damage and gastroduodenal diseases, studies evaluating gastric cancer patients are scarce. We investigated whether the functional *oip*A “on” status was associated with gastric cancer in the North-eastern Brazil, region with high prevalence of gastric cancer.

**Methods:**

We included samples from 95 *H. pylori* positive subjects (23 patients with gastritis, 24 with gastric cancer, 32 first-degree relatives of gastric cancer patients and 16 children). *oip*A was assayed by polymerase chain reaction (PCR) and DNA sequencing. *cag*A and *vac*A status were evaluated by PCR.

**Results:**

Overall 81.1% of the *H. pylori* strains had functional *oip*A*.* In adults, the *oip*A “on” status (OR = 9.20; 95%CI = 1.45–58.48, *P* = 0.02) and increasing age (OR = 1.08; 95%CI = 1.03–1.14; *P* = 0.003) were independently associated with gastric cancer in a logistic model. The *oip*A “on” status (OR = 14.75; 95%CI: 2.53–86.13, *P* = 0.003) was also associated with first-degree relatives of gastric cancer patients when compared with gastritis. The frequency of *oip*A “on” status did not differ between children and adults (*P* = 0.87). The *oip*A “on” status was significantly correlated with the presence of *cag*A and *vac*A s1 m1.

**Conclusion:**

*oip*A “on” status was independently associated with gastric cancer and first-degree relatives of gastric cancer patients in North-eastern Brazil.

## Background

Gastric cancer is the fifth most common cancer and the third leading cause of mortality among men and the fifth among women, with more than 700,000 deaths per year worldwide [1]. The malignancy is more frequently observed in developing than in developed countries [1, 2]. In Brazil, gastric cancer is the fourth most common malignancy among men and the sixth among women, excluding skin tumors [3]. In the North-east Brazil, one of the least developed regions of the country, gastric cancer is the second and the sixth most common tumor among males and females, respectively [3].

*Helicobacter pylori* is a well-recognized bacterium that chronically infects the stomach of approximately half of the world’s population being more prevalent in developing countries. Chronic *H. pylori* infection is considered the strongest risk factor for distal gastric adenocarcinoma [4]. The prevalence of *H. pylori* infection is estimated in approximately 90% of gastric cancer patients [1, 5]. In addition, the bacterium is associated with low grade B-cell MALT gastric lymphoma [6] and significantly increases the risk of development of peptic ulcer disease [7]. The mechanisms by which the infection progresses to the associated diseases are not completely known and depend on the relationship among host genetics, environmental and bacterial virulence factors [8].

Among the bacterial virulence genes, *cag*-PAI (cytotoxin associated gene pathogenicity island), containing several genes that trigger abnormal cellular signals, is considered the most important risk factor for *H. pylori*-associated gastric cancer. *vac*A virulence gene that encodes a vacuolating cytotoxin A (VacA) is also associated with *H. pylori* severe diseases; gastric cancer and duodenal ulcer [9].

Another group of putative virulence genes belongs to the *H. pylori* outer membrane proteins. OipA *(*outer inflammatory protein), one member of this large protein family, is encoded by *oip*A gene. The expression of the OipA is predicted to be regulated by a slipped strand mispairing system based on the number of CT dinucleotide repeats in the 5′ signal peptide coding region of the gene with “on” meaning that *oip*A is functional and “off*”* when the gene is non-functional [10]. *oip*A functional status is involved in the bacterial adherence to the gastric epithelial cells and in mucosal inflammation [11]. Moreover, the protein has been associated with interleukin (IL)-8 induction, mucosal damage and with duodenal ulcer [11]. A study evaluating Colombian patients demonstrated association of OipA with gastric cancer [12]. However, there are geographic variations. Studies from Italy and Netherlands have not demonstrated association between *oip*A and *H. pylori* clinical outcomes [13, 14]. To date, there are no studies evaluating the *oip*A functional status and risk of gastric cancer in Brazil. Moreover, it has to be emphasized that first-degree relatives of gastric cancer patients, who are thought to be at increased risk of gastric cancer, have not been evaluated yet. Therefore, the aim of the present study is to evaluate whether the functional *oip*A “on” status is associated with gastric cancer and first-degree relatives of gastric cancer patients in North-east Brazil, characterized by both high prevalence of gastric cancer and *H. pylori* infection.

## Methods

### Patients

The study was approved by the Ethics Committee of the University of Ceará and informed written consent was obtained from all adults and children (whenever possible) and their legal guardians.

We included 95 subjects infected with *H. pylori* strains: 23 with gastritis (13 females; mean age 45.6 ± 13.6 years; range 19–65 years) and 24 with non-cardia gastric cancer (9 females; mean age, 61.2 ± 15.4 years; range 36–84 years) who underwent upper endoscopy for evaluation of dyspeptic symptoms or underwent gastric surgery to remove gastric carcinoma at the University Hospital Walter Cantídio, Fortaleza, Brazil. Thirty-two *H. pylori*-positive first-degree relatives of gastric cancer patients (25 females; mean age 44.4 ± 10.2; range 19–60 years) attending the Walter Cantídio Hospital to be submitted to endoscopic screening for gastric cancer were randomly selected. DNA was also obtained from gastric juice/mucus by string test [15] from 16 asymptomatic children (6 girls; mean age, 12.6 ± 3.2 years; range 8–18 years) who had previously participated in a *H. pylori* epidemiological study in Parque Universitário, an urban community in Fortaleza, Brazil, from whom the *H. pylori* status was determined by ^13^C-urea breath test according to the protocol previously validated for the Brazilian population [16]. In the group of gastritis patients and first-degree relatives of gastric cancer patients, endoscopic biopsy samples were obtained from the antral and oxyntic gastric mucosa for histological, microbiological and DNA evaluation. Antral and oxyntic biopsy specimens were fixed in 10% formalin and embedded in paraffin wax, and 4-μm-thick histological sections were stained with carbolfuchsin for *H. pylori* investigation [17] and hematoxylin and eosin for histological analysis according to the updated Sydney System [18]. In the group of gastric cancer patients, the fragments were obtained from the stomach removed by gastrectomy after opening it along the greater curvature within 1 h of resection. The tumor was classified according to Laurén classification [19]. Gastric fragments were obtained from the gastric cancer patients (5 cm from the tumor) for microbiological, histological and DNA evaluation. Adults and children who had taken antimicrobials 30 days before and/or pump proton inhibitor 2 weeks before the procedures were excluded from the study.

### DNA extraction

The DNA was extracted using QIAmp® Kit (QIAGEN, Hilden, Germany) according to the manufacturer’s recommendations. The DNA concentration was determined by spectrophotometry using NanoDrop 2000 (Thermo Scientific, Wilmington, NC) and stored at − 20 °C until use.

### *oip*A genotyping and sequencing

The *oip*A gene was amplified by using the primers and thermo cycling conditions previously described by Yamaoka et al. [10]. Ninety-five *H. pylori oipA*-positive strains were PCR sequenced in order to assess the *oip*A status. The PCR products were purified using Wizard SV Gel® and PCR Clean-up System® (Promega, Madison, MI), and then the purified products were sequenced using the Big Dye Terminator kit version 3.1 Cycle Sequencing® in the ABI 3130 Genetic Analyzer® system (Applied Biosystems, Foster City, CA). The nucleotide sequences were analyzed using CAP3 software and the BLAST system (http://www.ncbi.nlm.nih.gov).

### *cag*A and *vac*A genotyping

The *cag*A gene was amplified as previously described [20]. PCR amplification of the *vac*A signal sequence and mid-region was performed according to Ashour et al. [21], by using the oligonucleotide primers described by Atherton et al. [22]. The LPB 1010 *H. pylori* strain (s1 m1 and *cag*A-positive) was used as a positive control. The standard, Tx30A *H. pylori* strain (s2 m2 *vac*A genotype and *cag*A-negative) and distilled water were both used as negative controls.

### Statistical analysis

The association of each variable, including age, gender and *oip*A “on” status with the *H. pylori*-associated diseases (dependent variable) was tested in univariate analysis. All variables with a *P*-value of 0.20 or less were included in the full model of logistic regression. Odds ratio (OR) and 95% confidence interval (CI) were used as an estimate of the risk. The Hosmer-Lemeshow goodness-of-fit test was used to evaluate the fit of models. Correlations were evaluated by Pearson or Spearman’s correlations. Data were analysed with the software SPSS for Windows, v. 17.0 (SPSS Inc., Chicago, IL). The level of significance was set at *P* ≤ 0.05.

## Results

### *oip*A status and demographic data

The *H. pylori-*specific *oip*A gene was successfully sequenced in all evaluated samples.

Overall, 81.1% (77/95) of the *H. pylori* strains sequenced had *oip*A “on” status and 18.9% (18/95) had status “off”. No difference was observed between the mean age of patients infected with *oip*A “on” status (44.29 SD 19.5 yrs) and age of those infected with *oip*A “off” (40.67 SD 20.89 yrs) of *H. pylori* (*P* = 0.48).

The number of CT repeat patterns ranged from five to nine (Table 1). The 6 CT pattern [76.6% (59/77)] was the most frequent CT dinucleotide repeats found among the *oipA* “on*”* status. The other *oip*A “on” status observed were: 1 + 4 CT [19.5% (15/77)] and 9 CT [3.9% (3/77)] repeat patterns. Among the *oip*A “off” status (*n* = 18), the following CT repeat patterns were found: 5 CTs (*n* = 4/22.2%), 7 CT (*n* = 11/61.1%), 8 CT (*n* = 2/11.1%), 9 CT (*n* = 1/5.6%). The CT pattern frequency according to the different *H. pylori* positive subjects are shown in Table 2.

**Table 1:** Signal-sequence coding region of *H. pylori oip*A observed in a Brazilian population

Sequence	CT repeats/Number	Gene status
ATGAAAAAAGCYCTCTTACTAACTCTCTCTCTCT---------------------CGTTTTGGCTC	6	on
M K K A L L L T L S L S F W L		
ATGAAAAAAGCTCTCTTACTAACTTTCTCTCTCT---------------------CGTTTTGGCTC	1 + 4	on
M K K A L L L T F S L S F W L		
ATGAAAAAAGCYCTCTTACTAACTCTCTCTCTCTCTCTCT----------GTTTTGGCTC	9	on
M K K A L L L T L S L S L S F W L		
ATGAAAAAAGCYCTCTTACTAACTCTCTCTCT----------------------- CGTTTTGGCT	5	off
M K K A L L L T L S L V L A		
ATGAAAAAAGCYCTCTTACTAACTCTCTCTCTCTCT---------------- CGTTTTGGC	7	off
M K K A L L L T L S L S R F G		
ATGAAAAAAGCYCTCTTACTAACTCTCTCTCTCTCTCT-------------CGTTTTGGCT	8	off
M K K A L L L T L S L S L V L A		
ATGAAAAAAACYCTCTTACTCTCTCTCTCTCTCTCT-----------------CGTTTTGGC	9	off
M K K A L L L S L S L S R F G

**Table 2:** Frequency of *oip*A “on” status in subjects (*n* = 118) with *H. pylori* associated diseases

n(CT)	CG (23)	GC (24)	GC rel (32)	Children (16)	Total (95)
	n (%)	n (%)	n (%)	n (%)	n (%)
On					
6	13 (56.5)	18 (75.0)	21 (65.6)	07 (43.8)	59 (62.1)
1 + 4	01 (4.3)	03 (12.5)	09 (28.1)	02 (12.5)	15 (15.7)
9	–	01 (4.2)	–	02 (12.5)	03 (3.2)
Total	14 (60.9)	22 (91.7)	30 (93.8)	11 (68.8)	77 (81.1)
Off					
7	06 (26.1)	02 (8.3)		03 (18.7)	11 (11.6)
8	01 (4.3)	–	01 (3.1)	–	02 (2.1)
5	02 (8.7)	–	01 (3.1)	01 (6.3)	04 (4.2)
9	–	–	–	01 (12.5)	01 (1.1)
Total	09 (39.1)	02 (8.3)	02 (6.2)	05 (37.5)	18 (18.9)

on, gene in frame; n, number; CT, cytosine-thymine repeats, off, gene out of frame; CG, chronic gastritis; GC, gastric cancer; GC rel, first-degree relatives of gastric cancer patients

**Fig. 1:**
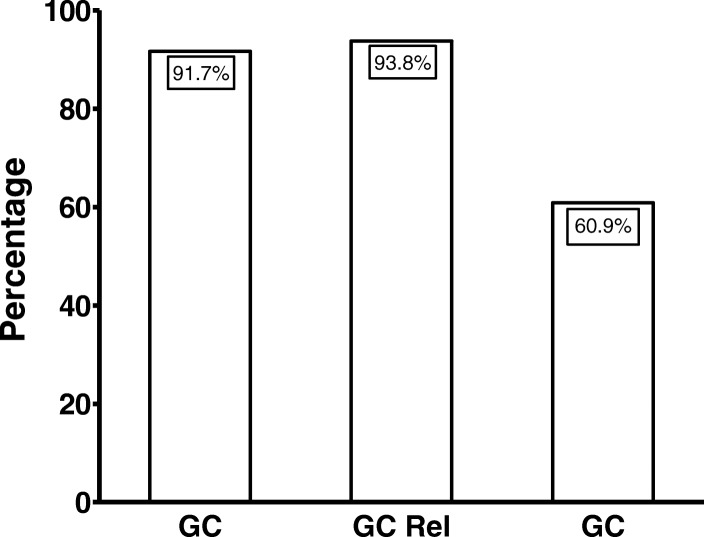
Frequency of *oip*A “on” status *Helicobacter pylori* strains in patients with gastric cancer (GC), first-degree relatives of gastric cancer patients (rel GC) and chronic gastritis (CG)

The functional “on” status was observed in 91.7% (22/24) of the adult patients with gastric cancer, in 60.9% (14/23) of the patients with gastritis and in 93.8% (30/32) of the first-degree relatives of gastric cancer patients (Fig. 1). Among children, the *oipA* “on” status was found in 68.8% (11/16) of the *H. pylori* strains, without significant difference from that of the adults with gastritis (*P* = 0.87; OR = 1.41; 95%CI = 0.37–5.55; two-tailed χ^2^-test).

### Association between *oip*A “on” status and gastric cancer

In order to identify variables independently associated with gastric cancer compared with gastritis, the data were analyzed in logistic regression model. In the univariate analysis, the *oip*A “on” status and increasing age were selected. In the multivariate analysis, the *oip*A “on” status and increasing age remained independently associated with gastric cancer (Table 3).

**Table 3:** Logistic regression models including gastric cancer or first-degree relative of gastric cancer patients as variables dependent in comparison with gastritis and *oip*A, age and gender as independent variables

Variables	Univariate	Multivariate		
	*P* value	OR	95% CI	*P* value
Gastric cancer^a^				
Increasing age	0.06	1.08	1.03–1.14	0.003
Gender	0.74			
*oip*A + status “on”	0.013	9.20	1.45–58.48	0.02
Relatives of GC^b^				
Increasing age	0.70	–	–	–
Gender	0.09	4.61	1.22–17.74	0.02
*oip*A + status “on”	0.003	14.75	2.53–86.13	0.003

+, positive; OR, odds ratio; CI, confidence interval; Relatives of GC, first-degree relatives of gastric cancer patients. The Hosmer-Lemeshow tests showed good fitness of the logistic regression model ^a^(*P* = 0.46; 8 degrees of freedom; 9 steps) and ^b^(*P* = 0.42; 8 degrees of freedom; 9 steps)

No difference was observed in the *oip*A “on” or “off” status and the tumor type. *oip*A “on” status was observed in 17/18 and in 5/6 intestinal and diffuse type tumor, respectively.

### Association between *oipA “*on*”* status and first-degree relatives of gastric cancer patients

Next, we evaluated the variables associated with the first-degree relatives of gastric cancer patients when compared with gastritis patients. In the univariate analysis, in addition to the *oip*A “on” status and increasing age, the gender was selected. In the multivariate analysis, *oip*A “on” statu*s* and gender remained associated with first-degree relatives of gastric cancer patients (Table 3).

### Comparison of *oip*A “on” status between gastric cancer and first-degree relatives of gastric cancer

No significant difference was observed in the frequency of *oip*A “on” status between gastric cancer and the first-degree relatives of gastric cancer patients (*P* = 1.0, two-tailed Fisher’s exact test).

### Correlation between *oip*A “on” status and other *H. pylori* virulent factors

In the gastritis group, the *oip*A “on” status was positively correlated with the presence of *cag*A (*r* = 0.27, *P* = 0.02) and *vac*A s1 m1 (*r* = 0.30, *P* = 0.01), but negatively correlated with the presence of *vac*A m2 allele (*r* = − 0.44, *P* = 0.001). In the gastric cancer patients, the percentage of simultaneously positive *oip*A “on” status and *cag*A was 91.7% (*r* = 1, *P* < 0.001). High positive correlation was also observed between in frame *oip*A and s1 m1 genotype (*r* = 0.80, *P* < 0.001). In respect to the gastric cancer relatives, high percentages were also observed in *oip*A “on” status and *cag*A positivity (88.0%) as well as in *oip*A “on” status and s1 m1 *vac*A genotype (83.3%).

### Association between *oip*A “on” status and histological gastritis

The presence of gastritis in the antral and oxyntic mucosa was higher in the first-degree relatives of gastric cancer patients (11–52.4%) than in the gastritis patients (3–13.0%), (a tendency of association; *P* = 0.08 – Mantel Haensel two-tailed chi-square test). In the group of gastritis patients, the pattern of the antral gastritis was more frequently mild than moderate/severe (4/9–44.4% vs. 5/19–26.3%). Otherwise, because the frequency of *oip*A “on” status was very high in the group of the first-degree relatives of gastric cancer patients, no association was observed between the presence of functional *oip*A and the degree of mononuclear and polymorphonuclear cells in the gastric mucosa.

## Discussion

Gastric cancer remains a major healthy problem, especially in developing countries. Unfavorable outcome of *H. pylori* infection to gastric cancer depends on genetics, environmental factors and virulence markers of the bacterium. Host genetic association has been demonstrated by the intra-familiar increased risk of gastric cancer. Notably, factors are emerging as key determinants. Among them, it has to be emphasized polymorphisms in genes involved in the adaptive immune response such as the IL-1β cytokine [23] and in members of the host’s innate immune response, Toll-like receptor-4 (TLR-4), which are associated with increased risk of gastric cancer [24]. In both cases the gastritis may progress to chronic atrophic gastritis, multifocal intestinal metaplasia and increased risk of gastric cancer. Unhealthy dietary patterns rich in starchy, meat, fat and high salt concentration contribute to gastric cancer development. In addition to well established association between gastric cancer and *cag*A and *vac*A here we confirmed functional *oip*A “on” status as a risk of gastric cancer and demonstrated that it is associated with first-degree relatives of gastric cancer patients. Furthermore, this study is the first in Brazil to evaluate the prevalence of *H. pylori oip*A “on” status in both adults and children. Overall the *oip*A “on” status was found in most of the evaluated strains (81.1%), similarly to that observed in Bulgaria (81.0%) [25], Colombia (79.3%) [13] and Venezuela (83.0%) [26]. It has to be emphasized that the prevalence of status “on” is very high in Asian countries such as Japan (100%) [27] and Malaysia/Singapore (> 85.0%) [28]. Otherwise, studies from developed Western countries such as Germany (59.0%) [29] and North Italy (60%) [30] demonstrated that the prevalence of *oip*A “on” status is lower than that observed in the current study. In children, the frequency of *oip*A “on” status was higher in the *H. pylori* strains from Brazil than in those from Portugal (49.6%) [31] and USA (45.9%) [32]. This high frequency of *oip*A “on” status observed in the children is in agreement with high prevalence of other *H. pylori* risk factors we have previously observed in children from the same population [15]. Taking together, the differences among countries point to regional differences that may be linked to differences in the social levels, genetics and/or environmental factors.

Remarkably, we observed that the functional *oip*A status was significantly associated with gastric cancer, even after adjusting for confounding factors as reported in Colombia [13], where the incidence of gastric cancer is similar to that observed in Ceará state, North-east of Brazil, and unlike in USA [11]. Furthermore, we observed that *oip*A “on” status was significantly more frequent in the first-degree relatives of gastric cancer patients than in those with gastritis. This finding is a novelty because we are not aware of studies evaluating *oip*A *H. pylori* virulence marker in such individuals who are at increased risk of gastric cancer that is determined by both bacterial and host factors. It has to be emphasized that first-degree relatives of gastric cancer patients are prone to be colonized by bacteria that circulate within a family. In fact, genetic fingerprint methods have demonstrated genetic homogeneity in the *H. pylori* strains within a family. In a previous study, we have shown that first-degree relatives of gastric cancer patients living in the same Brazilian region were colonized with *cag*A-positive and *vac*A more virulent *H. pylori* strains with the same characteristics of those isolated from their parents/siblings with gastric cancer [33].

Evidences that point to the *oip*A “on” as a gastric cancer risk factor include the ability of the bacterium carrying a functional *oipA* to attach to the gastric epithelial cells [12, 34], to induce inflammation [12, 34], apoptosis [34] and toxic effect towards cultured gastric epithelial cell lines [34, 35].

*H. pylori* gastric colonization induces epithelial cells to produce a series of pro-inflammatory and anti-inflammatory cytokines and chemokines; among them IL-8, a potent neutrophil chemotactic and activating peptide [10, 11, 36]. Of interest, Yamaoka et al. have demonstrated that the presence of *oip*A functional gene is associated with increased IL-8 production by gastric cancer cell line [10, 37] and with high concentrations of IL-8 in the antral mucosa [11].

Among the seven *oip*A sequencing patterns we detected, the 6 CT repeat pattern was the most common *oip*A “on” status found, in consonance with other studies in Western countries [11, 12, 24], but different from that observed in Asian countries where the *H. pylori* strains had less than 5 CT repeats in the *oip*A gene [27].

In this study, the *oip*A “on” status could not be discriminated from the other *H. pylori* virulent factors because all of them were highly frequent and closely linked to each other, especially the in frame *oip*A and *cag*A virulence factors that have been considered as having a synergistic effect on the pathogenesis induced by *H. pylori*.

Although the sample size had been enough to discriminate the results of high frequency of *oip*A “on” status between gastric cancer/first-degree relatives of gastric cancer patients and patients with gastritis alone, limitations of our study should be considered. An eventual bias is the small sample size that may preclude associations between *oip*A “on” status and age and gender. In addition, the small sample size impaired us to evaluate associations between the expression of *oip*A “on” status and scores of gastritis in the group of first-degree relatives of gastric cancer patients. Because, we evaluated *H. pylori* strains of people living in one of the least developed Brazilian regions, it is possible that these findings differ among the different regions of our country that have marked difference in the prevalence of *H. pylori-*positive status as well as in the prevalence of gastric cancer, which points to the need for further studies on this subject in other geographical regions.

## Conclusion

In conclusion, the *oip*A “on” status is associated with gastric cancer and with first-degree relatives of gastric cancer patients in North-eastern Brazilian population.

## Abbreviations

*cag*A: cytotoxin associated gene
CagA: Cytotoxin associated protein
*cag*-PAI: cytotoxin associated gene pathogenicity island
*H. pylori*: *Helicobacter pylori*
*oip*A: Outer inflammatory gene
OipA: Outer inflammatory protein
PCR: Polymerase chain reaction
*vac*A: vacuolating cytotoxin gene
VacA: Vacuolating cytotoxin protein
